# White Fibrous Papulosis of the Axillae and Neck

**DOI:** 10.7759/cureus.7635

**Published:** 2020-04-11

**Authors:** Yelena Dokic, Jaime Tschen

**Affiliations:** 1 Dermatology, Baylor College of Medicine, Houston, USA; 2 Dermatology, St. Joseph Dermatopathology, Houston, USA

**Keywords:** white fibrous papulosis of the neck, wfpn, fibroelastolytic papulosis of the neck, white fibrous papulosis of the axilla

## Abstract

Fibroelastolytic papulosis of the neck (FEPN) consists of two disorders: white fibrous papulosis of the neck (WFPN) and pseudoxanthoma elasticum-like papillary dermal elastolysis (PXE-PDE). The neck and supraclavicular areas are frequently involved; however, axillary involvement is significantly more rare, especially for white fibrous papulosis. Herein, we present an unusual case of white fibrous papulosis of the axillae, in addition to the neck, in a Caucasian woman.

## Introduction

White fibrous papulosis of the neck (WFPN) presents as many white, round, discrete, asymptomatic papules, approximately 2-3 mm in diameter, on the neck. It is a rare condition, first described in Japan, that tends to affect elderly individuals [1]. It belongs to the broader category of fibroelastolytic papulosis of the neck (FEPN). FEPN has two subcategories, pseudoxanthoma elasticum-like papillary dermal elastolysis (PXE-PDE) and WFPN, the latter of which is present in our patient [2]. It is rare for white fibrous papulosis to occur in the axillae, as it typically occurs on the neck, but such is the case for our patient. Herein, we present an unusual case of white fibrous papulosis of the axillae, in addition to the neck, in a 65-year-old Caucasian woman, which had been present for several years at the time of presentation. 

## Case presentation

**Figure 1:**
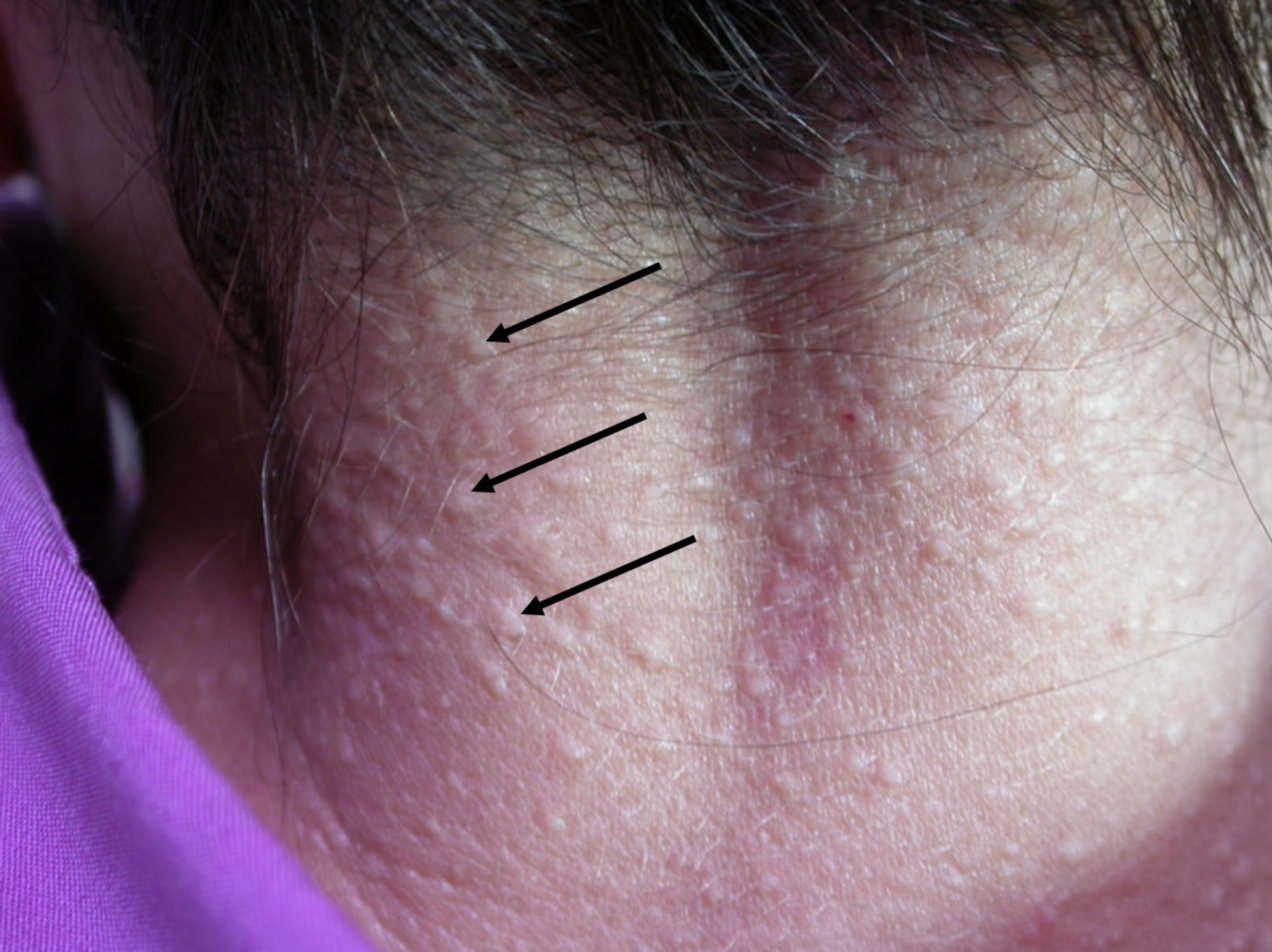
White fibrous papulosis of the neck The arrows indicate white fibrous papules.

**Figure 2:**
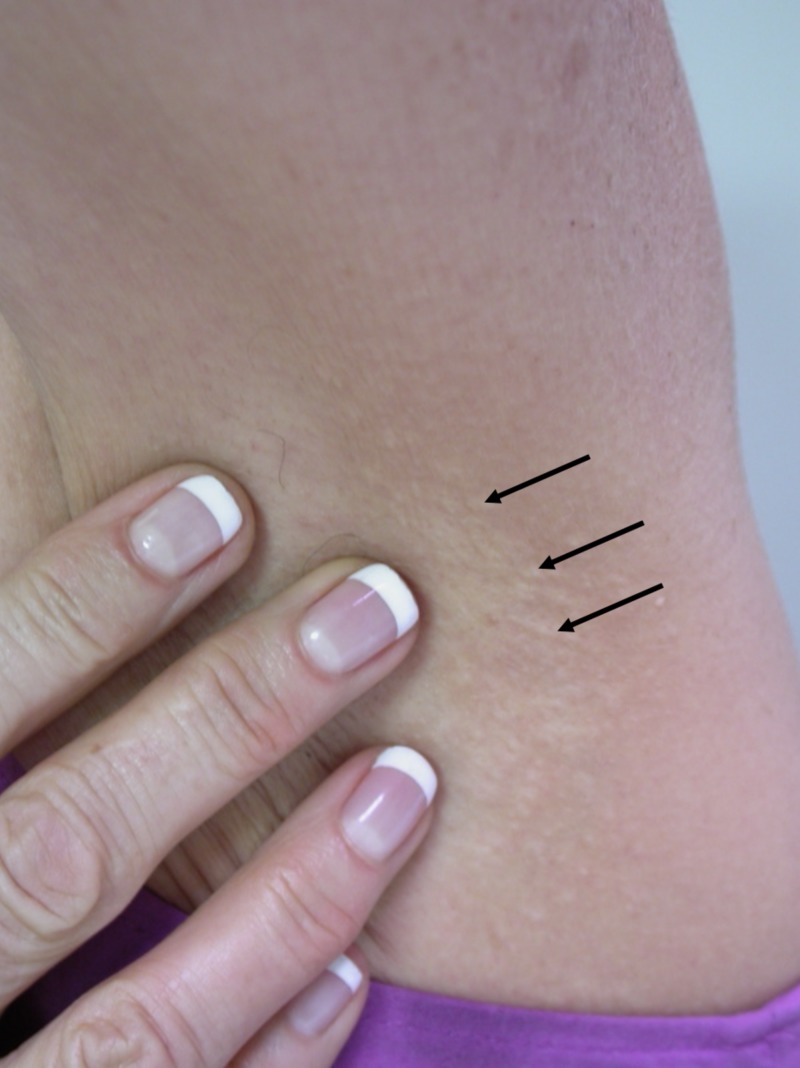
White fibrous papulosis of the axilla The arrows indicate white fibrous papules.

A 65-year-old woman presented with white papules on the posterior neck and her bilateral axillae that arose two years prior to presentation. She only noted occasional itchiness. No members of her family have had this condition. Additional history revealed no trauma to the area or irritants to the skin. The patient’s medical history was unremarkable. On examination, the patient was found to have numerous, 2-3 mm sized, small, white papules on her posterior neck (Figure 1), as well as bilateral axillae (Figure 2). No additional papules were located in any other region of the patient’s skin. She had not previously sought treatment for the lesions. The remainder of the physical exam was unremarkable.

**Figure 3:**
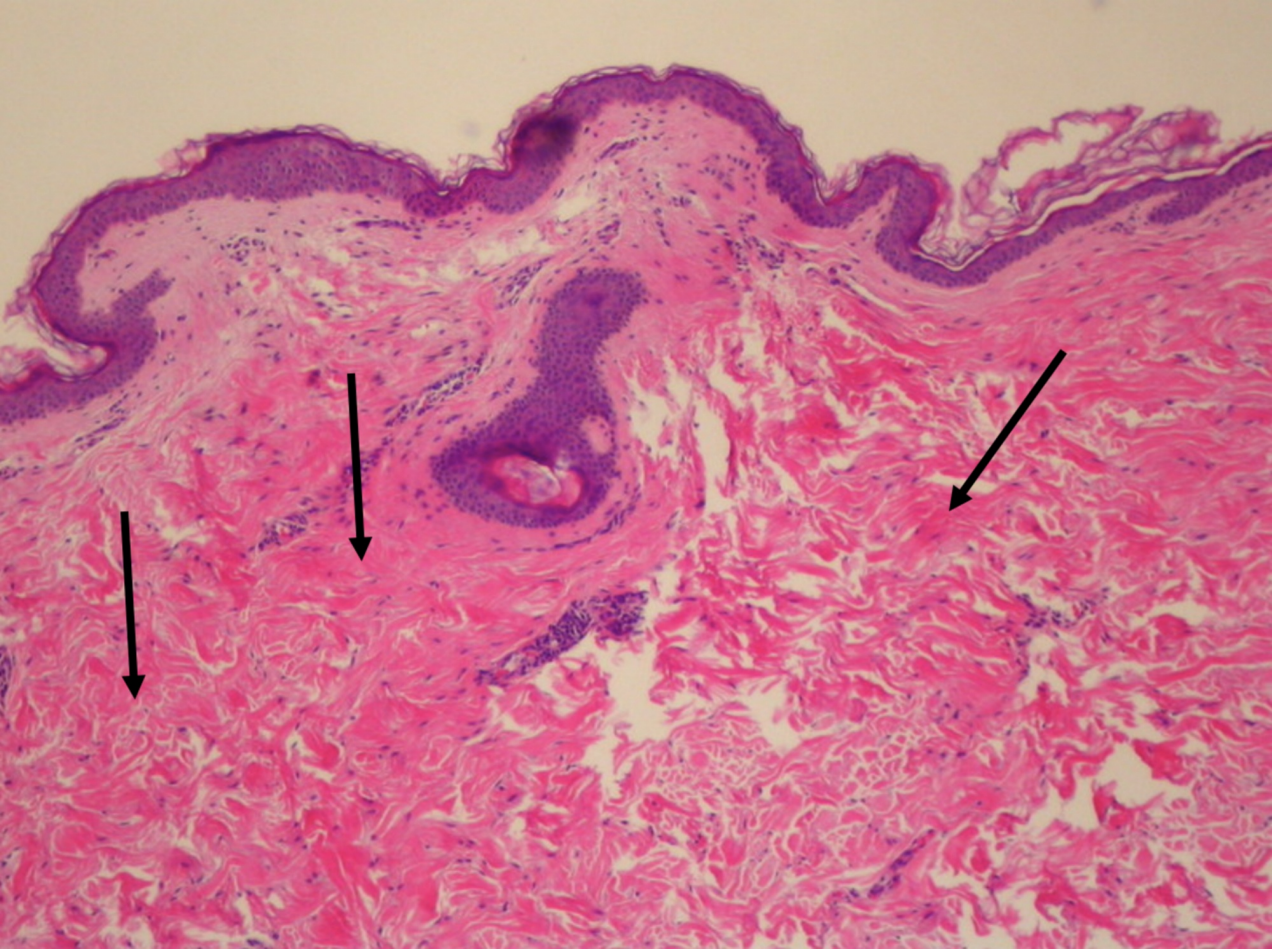
Dense fibrous tissue in dermis A 4-mm punch biopsy from the axilla. The arrows indicate well-circumscribed aggregates of dense fibrous tissue within the dermis. Hematoxylin and eosin stain, original magnification ×100.

**Figure 4:**
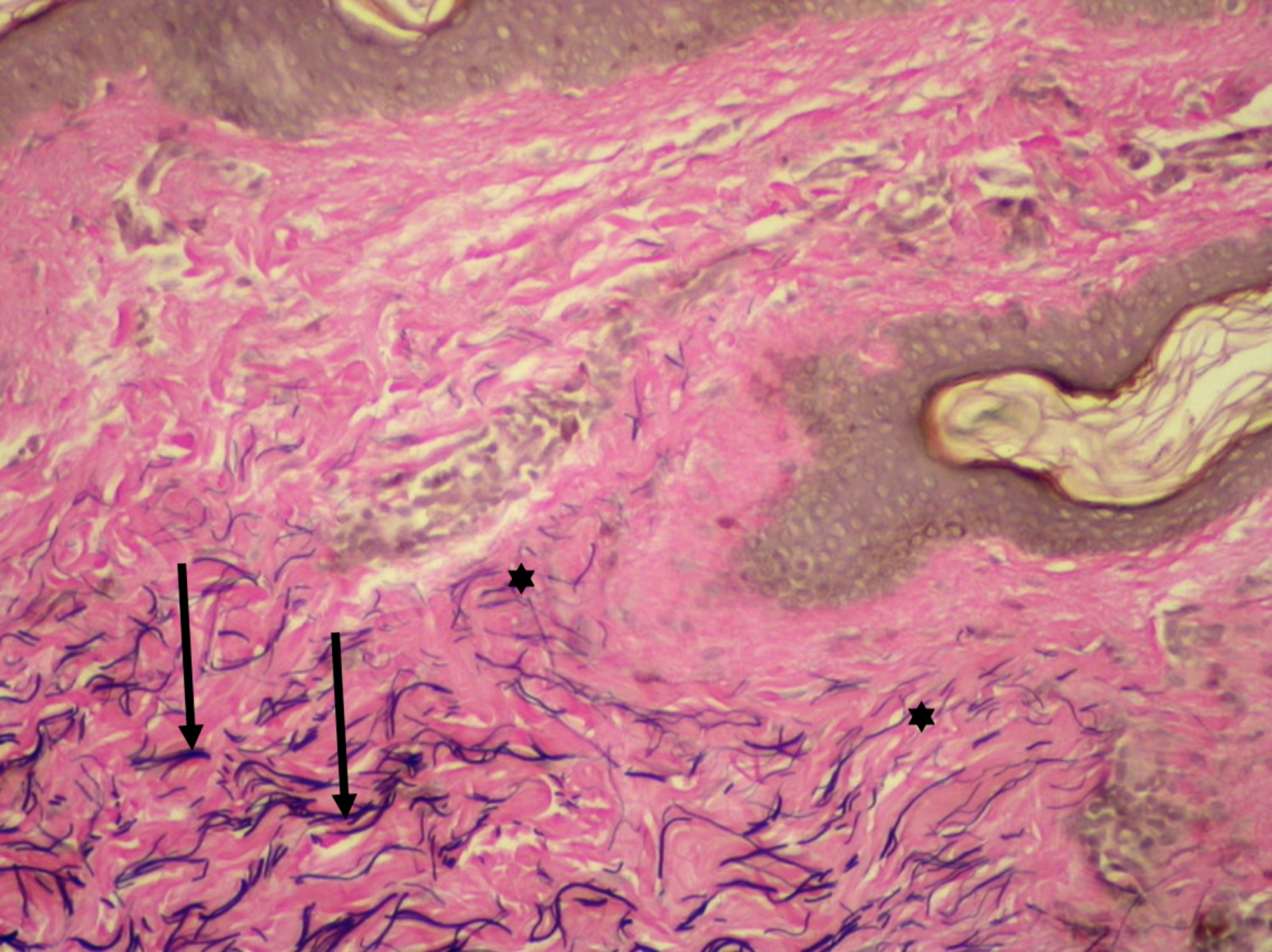
Elastolysis of papillary dermis A 4-mm punch biopsy from the axilla. The arrows indicate elastic fibers in the deeper dermis. The asterisks indicate elastolysis in the papillary dermis. Special stain (Verhoeff-van Gieson stain for elastic fibers), original magnification ×200.

A 4-mm punch biopsy of a white papule in the axilla was performed, and histopathology revealed well-circumscribed aggregates of dense fibrous tissue within the dermis (Figure 3). Special stain for elastic fibers using the Verhoeff-van Gieson stain revealed elastolysis of the papillary dermis (Figure 4). Biopsy and histological analysis confirmed the diagnosis of white fibrous papulosis.

The patient was counseled on the condition and declined further treatment. 

## Discussion

WFPN presents as many white, round, asymptomatic papules, approximately 2-3 mm in diameter, on the neck. It is a rare condition that tends to affect elderly individuals. Shimizu first described the disease in 1985 in Japan, and it tends to affect elderly Asians, particularly Japanese males [1]. 

WFPN is a subcategory that belongs in the more general category of FEPN. FEPN has two subcategories, PXE-PDE and WFPN, the latter of which is present in our patient. Briefly, PXE-PDE presents as soft yellow-white papules on the neck and supraclavicular areas, which can coalesce into plaques [2]. 

White fibrous papulosis tends to affect individuals in areas such as the neck, whereas PXE-PDE occurs in the neck, supraclavicular areas, antecubital fossa, and axillae [2]. It is highly rare for white fibrous papulosis to occur in the axillae, but such is the case for our patient. Affected individuals tend to be over 40 years of age, with increasing incidence in the elderly population. The lesions typically occur in Japanese males. Thus, it is very unusual for the papules to occur in Caucasian females, such as our patient [2]. 

During the aging process, the skin undergoes cutaneous aging due to both extrinsic and intrinsic factors. Extrinsic factors include elements such as excess sunlight exposure, which can lead to photoaging. Intrinsic factors include skin thinning, with thickening of collagen bundles [3]. PXE-PDE and WFRN are clinicopathological patterns of intrinsic aging of the skin. Although the exact pathophysiology of FEPN is unknown, it has been proposed that fibroblasts initiate subpapillary elastogenesis and cell activation in an attempt to counter the disappearance of elastic tissue [4]. This process could then potentially lead to the dermatologic manifestation of white papules.

Diagnosis is made by biopsy, which can confirm the clinical diagnosis of white fibrous papulosis. Histological examination of white fibrous papulosis reveals mild orthokeratotic hyperkeratosis, haphazardly arranged bundles of collagen fibers in the reticular and deep dermis, sparse superficial perivascular infiltrate, elastolysis, and elastic fibers with a normal or decreased diameter [5]. 

Because the lesions are persistent, and can occasionally be pruritic, patients sometimes seek treatment [6]. Various therapies for white fibrous papulosis have been attempted, with varying success. The use of topical tretinoin or topical antioxidant to reduce free-radical-induced aging has proven unsatisfactory [3]. If the lesions are well circumscribed, then surgical methods may be implemented [3]. There has also been a reported case of successful treatment of extensive papules with non-ablative fractional photothermolysis laser (fractionated 1550-nm Erbium Glass laser; Mosaic, Lutronic Co., Ltd, Seoul, South Korea) [5]. Excision or laser treatment is currently the best treatment option for white fibrous papulosis.

## Conclusions

Our patient was a 65-year-old woman who presented with white papules on the posterior neck and her bilateral axillae. A punch biopsy of a white papule in the axilla was performed, and histopathological examination confirmed a diagnosis of white fibrous papulosis. Due to the benign nature of the condition, the patient declined further treatment after being counseled on her condition. In conclusion, white fibrous papulosis of the neck can also occur, rarely, in the axillae.
